# Exploring Serum Levels of Brain Derived Neurotrophic Factor and Nerve Growth Factor Across Glaucoma Stages

**DOI:** 10.1371/journal.pone.0168565

**Published:** 2017-01-09

**Authors:** Francesco Oddone, Gloria Roberti, Alessandra Micera, Anna Busanello, Stefano Bonini, Luciano Quaranta, Luca Agnifili, Gianluca Manni

**Affiliations:** 1 IRCCS-Fondazione GB Bietti, Rome, Italy; 2 Department of Ophthalmology, Campus Bio-Medico University, Rome, Italy; 3 Section of Ophthalmology, Department of Medical and Surgical Specialties, Radiological Sciences, and Public Health University of Brescia, Brescia, Italy; 4 Ophthalmology Clinic, Department of Medicine and Aging Science, "G. d'Annunzio" University of Chieti-Pescara, Chieti, Italy; 5 DSCMT, University of Rome Tor Vergata, Rome, Italy; Massachusetts Eye & Ear Infirmary, Harvard Medical School, UNITED STATES

## Abstract

**Purpose:**

To investigate the serum levels of Brain Derived Neurotrophic Factor (BDNF) and Nerve Growth Factor (NGF) in patients affected by primary open angle glaucoma with a wide spectrum of disease severity compared to healthy controls and to explore their relationship with morphological and functional glaucoma parameters.

**Materials and Methods:**

45 patients affected by glaucoma at different stages and 15 age-matched healthy control subjects underwent visual field testing, peripapillary retinal nerve fibre layer thickness measurement using Spectral Domain Optical Coherence Tomography and blood collection for both neurotrophins detection by Enzyme-Linked Immunosorbent Assay. Statistical analysis and association between biostrumental and biochemical data were investigated.

**Results:**

Serum levels of BDNF in glaucoma patients were significantly lower than those measured in healthy controls (261.2±75.0 pg/ml vs 313.6±79.6 pg/ml, p = 0.03). Subgroups analysis showed that serum levels of BDNF were significantly lower in early (253.8±40.7 pg/ml, p = 0.019) and moderate glaucoma (231.3±54.3 pg/ml, p = 0.04) but not in advanced glaucoma (296.2±103.1 pg/ml, p = 0.06) compared to healthy controls.

Serum levels of NGF in glaucoma patients were significantly lower than those measured in the healthy controls (4.1±1 pg/mL vs 5.5±1.2 pg/mL, p = 0.01). Subgroups analysis showed that serum levels of NGF were significantly lower in early (3.5±0.9 pg/mL, p = 0.0008) and moderate glaucoma (3.8±0.7 pg/ml, p<0.0001) but not in advanced glaucoma (5.0±0.7 pg/ml, p = 0.32) compared to healthy controls.

BDNF serum levels were not related to age, visual field mean deviation or retinal nerve fibre layer thickness either in glaucoma or in controls while NGF levels were significantly related to visual field mean deviation in the glaucoma group (r^2^ = 0.26, p = 0.004).

**Conclusions:**

BDNF and NGF serum levels are reduced in the early and moderate glaucoma stages, suggesting the possibility that both factors could be further investigated as potential circulating biomarkers for the early detection of glaucoma.

## Introduction

Glaucoma is the leading cause of irreversible blindness in the world. It has been estimated that 60.5 million people were affected by primary open-angle glaucoma and primary angle-closure glaucoma globally in 2010 [1]. A recent meta-analysis estimates that the number of people with glaucoma worldwide will increase to 111.8 million in 2040 [2].

Glaucoma is a complex, multifactorial, neurodegenerative disease characterized by a progressive degeneration of retinal ganglion cells (RGCs) [3,4].

The pathophysiology of glaucomatous neurodegeneration is not fully understood [4]. Neuronal loss occurs largely by apoptosis, probably induced by a broad variety of stimuli, and possibly counteracted by neurotrophic factors (NTs), known to promote both neuronal development and survival troughout binding to specific receptors [5].

Evidences from both humans and experimental models suggest that intraocular pressure (IOP) elevation obstructs anterograde and retrograde axonal transport in RGCs axons at the level of the optic nerve head, leading to RGCs death [6,7].

Several studies indicate brain derived neurotrophich factor (BDNF), Nerve growth factor (NGF), ciliary neurotrophic factor (CNTF) and cell line derived neurotrophic factor (GDNF) as principally involved in RGCs survival [8].

BDNF is produced by RGCs and other accessory cells, and in turn plays a vital role in RGCs survival [9]. BDNF effects are mediated by binding to high-affinity receptor, tropomyosin receptor kinase B (TrkB) and the pan-neurotrophin p75^NTR^, constitutively expressed in the retina and lamina cribrosa [10].

The first discovered neurotrophic factor, NGF is produced and utilized specifically by RGCs, bipolar neurons and glial cells, and is thought to have crucial protective effects in several disease states [11].

Cells signaling occurs by means of the specific tyrosin kinase receptor trkA and the pan-neurotrophin receptor p75 (referred as p75^NTR^) [12].

While trkA mediates mainly cell growth, formation, elongation and regeneration of neuritis, p75^NTR^ leads to cell apoptosis or survival in different cellular contexts [12].

Lambiase and coworkers reported that NGF eye drops reduced RGCs loss in glaucomatous rats and that topical NGF treatment in three patients with advanced glaucoma improved all parameters of visual function [13].^.^

Furhtermore, Domenici and coworkers found that BDNF topical eye treatment recovered pattern elettroretinogram and visual evoked potential impairment increasing the number of Brn3 immunopositive RGCs in a glaucoma experimental model [14].

It has been demonstrated that both NGF and BDNF cross the blood brain barrier, with higher permeability for BDNF, compared to NGF [15].

Changes in NGF and BDNF levels in the serum have been reported for central neurodegenerative diseases such as Alzheimer’s disease and Parkinson’s [16,17].

Ghaffariyeh and co-workers reported a reduction of BDNF tear levels in normal tension glaucoma patients and a significant reduction of BDNF serum levels in early glaucoma [18,19].

No information is currently available regarding the serum levels of NGF in glaucoma patients and their relationship with functional and morphological biomarkers of the disease.

Therefore, the aim of this study was to evaluate the levels of NGF and BDNF in serum of patients affected by primary open angle glaucoma (POAG) with a broad spectrum of disease severity, in comparison to healthy controls and to explore their relationship with morphological and functional markers of disease severity.

## Materials and Methods

### Study population

This study has been approved by the ethics committee of IRCCS-Fondazione GB Bietti (Prot. CE 435/14) and of University of Rome Tor Vergata (Registro sperimentazionei 131.14), Rome, Italy.

Patients were recruited at University Campus Biomedico, at University of Rome Tor Vergata, and at IRCCS-Fondazione GB Bietti (Rome, Italy).

The study was then conducted at the IRCCS-Fondazione G.B.Bietti and at the University of Rome Tor Vergata, in accordance with the Declaration of Helsinki. Only patients older than 18 years old of both genders, and able to understand and sign the written informed consent were enrolled.

Two groups were evaluated for the study: POAG patients group and healthy control-subjects group. Forty-five patients were included in the POAG group (mean age 66±11.3 years, 24 males and 21 females) and 15 subjects were included in the control-subjects group (62.2±10.3 years, 6 males and 9 females, mean age).

Inclusion criteria for the glaucoma group were: history of IOP greater than 22 mmHg in at least two occasions, open-angle on gonioscopy, the presence of a repeatable visual field (VF) defect (as defined below), corresponding with optic nerve and RNFL damage as evaluated ophthalmoscopically by two independent expert investigators (F.O. and G.R.), and confirmed by the presence of one or more corresponding peripapillary retinal nerve fibre layer (RNFL) sectors outside normal limits by Spectral Domain Optical Coherence Tomography, SD-OCT, (Spectralis OCT, Heidelberg Engineering, Heidelberg, Germany).

A glaucomatous VF loss was defined as two consecutive reliable visual fields with glaucoma hemifiled test outside normal limits, mean deviation (MD) and pattern standard deviation with p<0.05, and a cluster in the pattern standard deviation plot of at least 3 points with p<0.05, one of each with p<0.01, not contiguous with the blind spot and not crossing the horizontal midline.

The reliablity indices considered were as follows: false positive <15%, fixation losses and false-negative responses <25%.

Glaucoma patients were stratified according to the VF defect as follows: early glaucoma (15 patients with MD, < -6dB), moderate glaucoma (15 patients with -6dB>MD<-12dB), advanced glaucoma (15 patients with MD>-12dB).

Patients with secondary glaucoma were excluded (Pigmentary glaucoma, Exfoliative glaucoma, Steroid-induced glaucoma).

Healthy controls age/sex matched had to have an IOP less than 22 mmHg, open-angle on gonioscopy, normal optic disc and VF test.

Participants in both groups were excluded if they had spherical refractive error greater than ±6 diopters, astigmatism greater than ±3 diopters, retinal diseases (including diabetic retinopathy or age-related macular degeneration), other optic neuropathies different than glaucoma, opacities of optic media that could bias functional and structural testing, active inflammatory or infective diseases, metabolic, autoimmune, neurological or neurodegenerative diseases, blood coagulation diseases, pregnancy or breastfeeding.

### Ophthalmological examination

Preliminary tests were performed to determine subject eligibility to be enrolled in the study. These tests included a comprehensive ophthalmological examination: slit lamp evaluation, gonioscopy, central corneal thickness and axial length measurements, VF testing by Humphrey Field Analyzer using the 24–2 Swedish Interactive Threshold Algorithm Standard test (Carl Zeiss Meditec, Dublin, CA), IOP measurement using Goldmann applanation tonometry, and indirect dilated ophthalmoscopy with a 90 diopters lens.

All patients had peripapillary RNFL thickness measurements performed using the Spectralis SD-OCT circular scan pattern. The scan circle around the optic nerve is approximately 3.5–3.6 mm in diameter. Scans were acquired in High Speed mode. The Spectralis OCT software (version 4.0) allows for automatic segmentation of the anterior and posterior borders of the RNFL to calculate the average RNFL thickness for the overall global (360 degrees) and for four quadrants superior [S], inferior [I], nasal [N], and temporal [T]). The average RNFL thickness measurement was used in the analysis.

Informations regarding the ocular hypotensive treatments and systemic treatments were also collected and are reported in Table 1.

**Table 1:** number of patients under ocular hypotensive treatments and systemic treatments in the whole study population.

	Early Glaucoma	Moderate Glaucoma	Advanced Glaucoma	Healthy Controls
**Carbonic anhydrase inhibitors**	3	6	10	0
**Prostaglandin analogues**	15	12	10	0
**β-Adrenergic receptor antagonists**	7	8	12	0
**α2-Adrenergic receptor agonists**	2	0	4	0
**Systemic β blockers**	3	2	5	4
**ACE inhibitors**	1	3	4	2
**Ca antagonists**	2	4	5	2
**Statins**	2	3	6	4
**Proton pump inhibitors**	3	2	6	4

### Blood specimens

At the clinical units, participants underwent peripheral blood sample collection between 7 and 10 am in vacutainer tubes (PBI International, Milan, Italy), left at room temperature for 30 min,subject to centrifugation (3500g for 10 min) to collect serum and delivered to the Laboratory according to standard procedures.

In the lab, samples were further centrifuged to remove eventual red cells and aliquotes were short-term stored at -70°C until processing.

Samples were analyzed within 3 months from sampling.

### NGF and BDNF assays

For NGF quantification, a 2-site NGF immunosorbent assay (0.5 pg/mL sensitivity) was performed by following the Weskamp and Otten procedure (1987), with minor modification [20,21]. Sera were diluited 1:2 in assay diluent (10mM PB, 150mM NaCl, 0.5%BSA, 0.1% Triton X100 and 1x protease inhibitor cocktail; ph7.5). In brief, 96-well Maxisorp enzyme-linked immunoassay plates (Nunc, Roskilde, Denmark) were precoated with monoclonal anti-human βNGF antibodies (0.4 μg/mL; MAB256; R&D Systems Inc, Minneapolis, Minnesota). Standards (0.15 pg/mL to 1 ng/mL 2.5 S βNGF, mature active NGF; Alomone Labs, Jerusalem, Israel) and samples were incubated at 4°C for 18 hours. Enzyme-linked immunosorbent assay (ELISA) was developed by using polyclonal biotinylated anti-human NGF antibodies (0.15 μg/mL, BAF256; R&D System), horseradish peroxidase streptavidin (1:300; DY998, R&D System) and the ready-to-use 3,3′,5,5′-TetraMethylBenzidine (TMB) substrate (eBioscience, San Diego, CA, USA). Colorimetric signals (OD 490–560nm) were quantified using an ELISA plate reader (Sunrise; Tecan Group Ltd., Männedorf, Switzerland). The data were normalized to total protein content as assessed at the beginning with the NanoDrop spectrophotometer (A280; Sunrise; Thermo-Fisher Scientific, Inc., Waltham, MA, USA). Under these conditions, no cross reactivity with BDNF or Neutrophins 3/4/5 was observed. BDNF was quantified using a commercially available high-sensitive ELISA (code DY248; sensitivity 20pg/ml; R&D System), according to manufacturer’s suggestions with minor modifications. Colorimetric signals of total protein quantification and data production were acquired as above. For NGF and BDNF ELISA, assays were performed in duplicate for each sample.

### Statistical analysis

Normality distribution of the data was checked by the Shapiro–Wilk test.

Descriptive analysis was expressed as mean and standard deviation (SD).

Differences in mean values between groups were evaluated by one-way analysis of variance (ANOVA) followed by post-hoc Student-Newman-Keuls test for all pairwaise comparisons. The differences between BDNF and NGF serum levels of each glaucoma subgroup and healthy controls are expressed as mean, standard deviation and 95% Confidence Interval (CI) for the mean.

Linear regression analysis was used to explore the relationships between biochemical (BDNF and NGF) and clinical (demographic and ophthalmic) variables. A p value <0.05 was considered statistically significant.

Statistical analysis was performed using MedCalc program (MedCalc Software INC., Mariakerke, Belgium, version 10.0.2.0).

Considering a SD of 40 pg/ml and a type 1 error of 0.05, a sample size of 14 patients in each group was calculated to obtain a power of 80% to detect a difference of 45 pg/ml of BDNF between groups.

## Results

Forty-five glaucoma patients and 15 healthy controls were included in the study. Glaucoma patients were categorized according to VF defect (15 patients with early glaucoma, MD<-6dB; 15 patients with moderate glaucoma, -12dB<MD>-6dB; 15 patients with advanced glaucoma MD>-12 dB). Demographics and clinical characteristics of the study population are reported in Table 2.

**Table 2:** Demographic characteristics of study population and results of ANOVA analysis to compare each glaucoma severity subgroups and healthy controls.

	*Glaucoma Total Mean±SD (p values vs controls)*	*Early Glaucom Mean±SD (p values vs controls)*	*Moderate GlaucomaMean±SD (p values vs controls)*	*Advanced Glaucoma Mean±SD (p values vs controls)*	*Healthy Controls Mean±SD (p values vs controls)*	p*
N	***45***	***15***	***15***	***15***	***15***	***Anova***
***Age (years)***	66±11.3 (0.25)	60.5±12.9	65.7±9.9	71.9±8.3 **(0.009)**	62.2±10.3	0.02
***M/F***	24/21	10/5	7/8	7/8	6/9	
***AL (mm)***	24.1±1.7 (0.52)	24.5±1.5	24.7±2.3	23.4±1.1	23.8±1.4	0.15
***IOP (mmHg)***	14.8±2.5 (0.19)	14.8±2.4	14.8±2.1	14.9±3.2	13.9±1.7	0.63
***CCT (μm)***	523.3±37.5 (0.23)	522.3±44.3	530.2±29.9	516.6±42.9	535.8±26.5	0.5
***MD (dB)***	-11.5±8.6 **(<0.001)**	-3.5±1.5 **(<0.0001)**	-8.6±1.5 **(<0.0001)**	-22.4±4.7 **(<0.0001)**	-0.7±1.7	**<0.001**
***PSD (dB)***	7.4±4 **(<0.001)**	3.5±1.8	8.5±3.6 **(<0.0001)**	10.2±2.7 **(<0.0001)**	2.1±0.9	**<0.001**
***RNFL (μm)***	63±14.5 **(<0.001)**	68.2±13.6 **(<0.0001)**	65.5±13.9 **(<0.0001)**	55.3±13.5 **(<0.0001)**	95±10.8	**<0.001**
***BDNF (pg/ml)***	261.2±75.0 **(0.03)**	253.8±40.7 **(0.019)**	231.3±54.3 **(p = 0.04)**	296.2±103.1	313.6±79.6	**0.02**
***NGF (pg/ml)***	4.1±1 **(0.001)**	3.5±0.9 **(0.0008)**	3.8±0.7 **(<0.0001)**	5.0±0.7	5.5±1.2	**<0.001**

Data are expressed as mean ± standard deviation. p values of statistically different groups compared to healthy controls are in bold.

AL = axial length; IOP = intraocular pressure; CCT = central corneal thickness; MD = mean deviation; PSD = pattern standard deviation; RNFL = retinal nerve fibre layer; BDNF = brain derived neurotrophic factor; NGF = nerve growth factor

Particularly, no differences were found in axial length (p = 0.52), central corneal thickness (p = 0.23) and IOP (p = 0.19) between glaucoma patients and healthy controls.

As expected, the visual field defect was worst in glaucoma patients compared to the healthy controls (p<0.001) as well as RNFL was thinner in the advanced stage of the disease compared to the early and moderate stages (p<0.001) and controls (p<0.001).

BDNF serum levels were statistically reduced in glaucoma groups when compared to healthy controls (261.2±75.0 pg/ml vs 313.6±79.6 pg/ml, p = 0.03). Similarly, NGF serum levels were stastistically reduced in the whole glaucoma population when compared to the healthy controls (4.1±1 pg/ml vs 5.5±1.2 pg/ml, p = 0.01).

**Fig 1:**
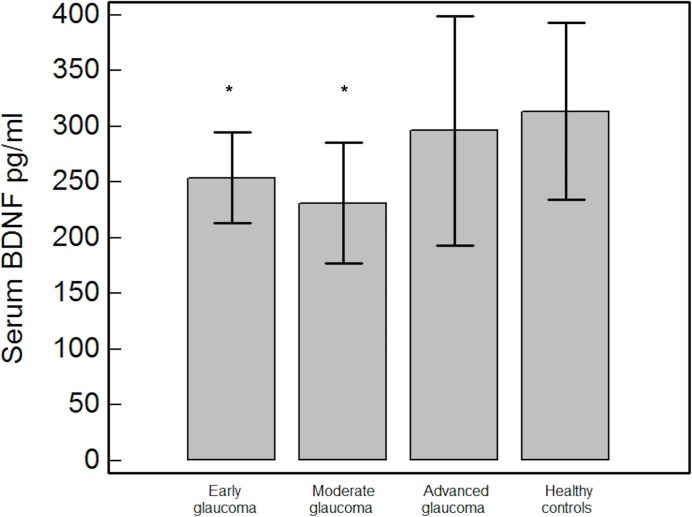
Comparison of BDNF serum levels between early glaucoma patients, moderate glaucoma patients, advanced glaucoma patients and healthy controls. BDNF = brain derived neurotrophic factor. * = p<0.05 for the post-hoc comparisons of each glaucoma severity subgroup and healthy controls.

Subgroups analysis showed BDNF levels were significantly lower in early (253.8±40.7 pg/ml, p = 0.019) and moderate glaucoma groups (231.3±54.3 pg/ml, p = 0.04) but not in advanced glaucoma group (296.2±103.1 pg/ml, p = 0.06) when compared to the control group (**Fig 1**).

**Fig 2:**
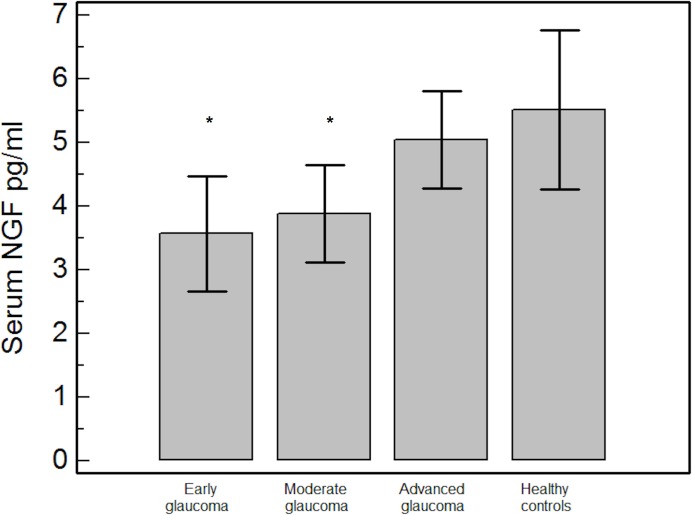
Comparison of NGF serum levels between early glaucoma patients, moderate glaucoma patients, advanced glaucoma patients and healthy controls. NGF = nerve growth factor. * = p<0.05 for the post-hoc comparisons of each glaucoma severity subgroup and healthy controls.

As well, NGF serum levels were significantly lower in early (3.5±0.9 pg/ml, p = 0.0008) and moderate glaucoma groups (3.8±0.7 pg/ml, p<0.0001) but not in the group of advanced glaucoma (5.0±0.7 pg/ml, p = 0.32) when compared to healthy controls (**Fig 2**).

The mean, the standard deviation and the 95%CI for the mean of the differences between BDNF and NGF serum levels in each glaucoma subgroup and BDNF and NGF serum levels in the control group are reported in Table 3 and Table 4.

**Table 3:** mean, standard deviation and 95%CI for the mean of the differences between BDNF serum levels of each glaucoma subgroup and healthy controls.

BDNF early-BDNF healthy	BDNF moderate-BDNF healthy	BDNF advanced-BDNF healthy
-59.7±88.3 pg/ml (-110.7 to -8.7)	-82.3±104 pg/ml (-142.3 to -22.2)	-17.3±140.2 pg/ml (-98.3 to 63.5)

**Table 4:** mean, standard deviation and 95%CI for the mean of the differences between NGF serum levels of each glaucoma subgroup and healthy controls.

NGF early-NGF healthy	NGF moderate-NGF healthy	NGF advanced-NGF healthy
-1.9±1.3 pg/ml (-2.8 to -1.0)	-1.6±1.3 pg/ml (-2.5 to -0.6)	-0.4±1,2 pg/ml (-1.3 to 0.4)

Hypotensive treatments are reported in Table 1. Three patients in the advanced group have been surgically treated, thus they were not under any topical hypotensive therapy.

### BDNF, NGF and age

Patients with advanced glaucoma were statistically older with respect to early glaucoma and healthy subjects (**Table 1**).

BDNF and NGF serum concentrations were not related to age in the whole glaucoma population (*BDNF/age* r^2^ = 0.004, p = 0.65; *NGF/age* r^2^ = 0.10, p = 0.08) nor in controls (*BDNF/age* r^2^ = 0.03, p = 0.52; *NGF/age* r^2^ = 0.02, p = 0.67).

Both NTs were not related to age either in all glaucoma subgroups (*BDNF/age*: early glaucoma r^2^ = 0.02, p = 0.59; moderate glaucoma r^2^ = 0.25, p = 0.06; advanced glaucoma r^2^ = 0.01, p = 0.65; *NGF/age*: early glaucoma r^2^ = 0.11, p = 0.33; moderate glaucoma r^2^ = 0.001, p = 0.92; advanced glaucoma r^2^ = 0.006, p = 0.81;).

### BDNF, NGF and MD values

In the whole glaucoma population, BDNF serum levels were not statistically related to MD values (r^2^ = 0.08, p = 0.06), while NGF serum levels were statistically related to MD values (r^2^ = 0.26, p = 0.004). Since MD values were statistically related to age (r^2^ = 0.15, p = 0.008), the linear regression analysis between both NTs and MD was adjusted for age and no statistically significant relationship was found (BDNF/MD adjusted for age r^2^ = 0.11, p = 0.27; NGF/MD adjusted for age r^2^ = 0.31, p = 0.28).

In healthy controls neither BDNF serum levels (r^2^ = 0.23, p = 0.08) nor NGF serum levels (r^2^ = 0.09, p = 0.4) were related to MD values and age was not related to MD values (r^2^ = 0.02, p = 0.5).

### BDNF, NGF and RNFL values

Both BDNF and NGF serum levels were not statistically related to RNFL thickness in the whole glaucoma sample (BDNF/RNFL r^2^ = 0.0002, p = 0.9; NGF/RNFL r^2^ = 0.01, p = 0.48) and RNFL thickness was not statistically related to age (r^2^ = 0.01, p = 0.44). As well, in healthy controls both BDNF and NGF serum levels were not related to RNFL thickness (BDNF/RNFL r^2^ = 0.004, p = 0.8; NGF/RNFL r^2^ = 0.11, p = 0.22) and RNFL thickness was not statistically related to age (r^2^ = 0.11, p = 0.22).

### BDNF, NGF and gender

Twenty-four males and 21 females were included in the whole glaucoma group. Both NGF and BDNF serum levels were higher in females compared to males but the differences were not statistically significant (BDNF: 263.53±97.5 pg/ml in females vs 247.22±71.74 pg/ml in males, p = 0.53. NGF: 4.37±0.93 pg/ml in females vs 4±1.09 pg/ml in males, p = 0.33).

Six males and 9 females were included in the healthy controls group. BDNF serum levels were higher in females compared to males (324.45±87.48 pg/ml vs 299.23±72.97 pg/ml), but the difference was not statistically significant (p = 0.57). Also NGF serum levels were higher in females when compared to males (5.86±1.11 pg/ml vs 4.97±1.39 pg/ml) but this difference was not statistically significant (p = 0.29).

## Discussion

This study explored levels of NGF and BDNF in serum of glaucoma patients with different stages of the disease in comparison with healthy controls.

To the best of our knowledge this is the first time NGF serum levels have been investigated in glaucoma patients, while serum levels of BDNF have already been reported in a previous investigation [19].

In that study, authors included twenty-five patients with early glaucoma defined according to the Hodapp-Parrish-Anderson grading scale of severity of VF defect, and found significantly reduced serum levels of BDNF compared to 25 healthy controls.

Herein, we included 45 glaucoma patients categorized as mild, moderate and severe glaucoma, according to the VF MD and 15 healthy controls. We found that both BDNF and NGF levels were significantly reduced in glaucomatous sera and particularly in patients with early and moderate glaucoma compared to healthy controls. Interestingly, we found similar serum levels of BDNF and NGF in advanced glaucoma patients and healthy controls.

The similar pattern of expression between BDNF and NGF in glaucomatous sera is actually with no explanation.

It has been reported that both NGF and BDNF are important neurotrophic factors produced by different cell types, released into bloodstream, and exerting crucial survival effects on adult neurons of the central nervous system [9].

A current hypothesis is NGF and BDNF are involved in the pathogenesis of RGCs death in glaucoma. Experimental studies have shown blockade of axonal transport induced by IOP elevation leads to deficits of the levels and availability of these factors and subsequent RGCs death [6,7]. In addition, changes in neurotrophin receptor expression as a result of elevated IOP could alter the effects of neurotrophins on RGCs survival [22]. As a matter of fact, it has been reported that the ratio of trkA to p75^NTR^ in the retina and optic nerve is shifted in favor of p75^NTR^, and so toward apoptosis, by IOP elevation [22].

The finding of higher serum levels of both NGF and BDNF in patients with advanced stage of the disease when compared to patients with early and moderate glaucoma, and similar to the levels found in healthy control subjects is matter of speculation.

It could be hypothesized the existence of compensatory survival/repair mechanisms that only take place in the advanced stages of the neurodegeneration. These mechanisms have already been suggested in studies evaluating the levels of neurotrophins in the serum of patients with Alzheimer’s disease [23–27]. Some authors observed a reduction while others reported an increase in serum levels of neurotrophins [23–27]. Laske et al. investigated the influence of different Alzheimer’s disease stages on BDNF serum concentrations, and observed that in patients with early stages an increased BDNF serum concentration with respect to more severe stages of Alzheimer’s disease and to the healthy controls [23]. These results supported the hypothesis of an upregulation of BDNF as a compensatory mechanism against neurodegeneration, as proposed in the study by Faria et al. [24]

However, Angelucci et al. measured BDNF serum levels in Alzheimer’s disease patients with several degrees of severity, mild cognitive impairment and healthy subjects, and found that the observed BDNF serum levels increase was neither dependent on treatment nor on disease severity. [25]. These findings were consistent with post-mortem examinations of Alzheimer’s disease brains. Infact, a significant increase of BDNF and the receptor TrkB has been shown in hippocampus,parietal cortex, astrocytes and senile plaques [26]. These authors defined the increase in BDNF as a compensatory repair mechanism both in early and late neurodegeneration. Furthermore, an increased NGF concentration in Alzheimer’s disease cortex was found when compared to non-demented controls [27].

Neverthless, the main culprit in Alzheimer’s disease is the precursor form of NGF, pro-NGF that binds to a sortilin-p75^NTR^ complex and activates cell death through the stimulation of Jun N-terminal kinase [28]. It’s been demonstrated that pro-NGF is copious in brains of patients with Alzheimer’s disease and this increase may reflect either an active role for proNGF or posttranslational disorders in NGF biosynthesis that reduce the processing of pro-NGF to NGF in Alzheimer’s disease [29].

It would be interesting to explore the role of pro-NGF in glaucoma disease too.

Since the ‘90s, glaucoma has been considered a disease of the central nervous system exhibiting several common mechanisms of cell death with neurodegenerative progressive diseases, such as Alzheimer’s disease [30]. We can therefore suppose that the increased serum levels of BDNF and NGF observed in patients with advanced glaucomatous damage compared to those of patients with early and moderate glaucoma and similar to those of healthy subjects could be explained with the same compensatory repair model described in patients with advanced Alzheimer’s disease [26,27].

Nevertheless it has to be considered that serum levels of neurotrophic factors can be influenced by a number of other systemic factors beside the presence of a neurodegenrative disease. Both in models and humans, stress, fear and anxiety are associated with enhanced rather than lowered serum levels of BDNF and NGF [31–33]. In order to minimize these systemic effects and to take in account a possible circadian rhythm of both NTs, blood samples were collected between 7 and 10 am and patients were advised to avoid any physical exercise before the visit. However, advanced glaucoma, causes visual impairment and affect different field of daily life reducing its quality and generating anxiety and stress that can’t be avoided [34]. Furthermore, the presence of comorbidities can also exacerbate these feelings.

One may retort that even depression is very common among old patients with chronic diseases, and that several studies report reduced level of both BDNF and NGF serum levels in major depressive disorder which contrast our results [35–37]. This is clearly true. However, a compensatory mechanism of increased BDNF has been described also to explain the improvement of the depressive symptoms [38]. Additionally the administration of antidepressant selective serotonin reuptake inhibitors have been shown to enhance BDNF gene expression [39]. A meta-analysis by Chen et al., instead, didn’t find any significant change of NGF serum level before and after adequate treatment for depression [40]. Patients with a confirmed diagnosis of major depression or taking any antidepressant drugs were excluded from the present study despite the presence of milder forms of latent depression cannot be excluded.

It would be very interesting to correlate the duration of the disease with the levels of serum neurotrophins in order to better clarify the relationship between the progression of the disease and the variation of serum NTs levels. Nevertheless in a cross-sectional study, like the present study, the duration of the disease is uncertain since one may rely only on the patients’ report which is mainly the time of diagnosis and thus not necessarily representative of the duration of the disease. In fact an early and an advanced glaucoma might be diagnosed at the same time despite very different disease duration. A prospective long-term cohort study might probably represent a good study design to assess how serum neurothrophins levels vary across time and to explore whether any compensatory mechanisms take place during the course of the disease at the more advanced stages.

When exploring the relationships of BDNF serum levels with functional glaucoma parameter we found no significant association in the whole sample glaucoma population while NGF serum levels were statistically related to the visual field mean deviation. The linear regression analysis between both NTs and MD was adjusted for age and no significant relationship was found. Both BDNF and NGF were not even related to the morphological glaucoma parameter (RNFL).

A negative correlation between plasma BDNF levels and age was described in a population of 140 healthy volunteers between 20 and 60 years of age [41] and BDNF decreased levels were confirmed in an older sample population (range 70–103 years) [42]. By contrast, in another study with 206 healthy subjects with a mean age of 44.3±13.30 (range 20–70) years, no age-related changes were found in circulating [43].

The age of our sample population ranged from 31 to 86 years and, in particular, patients with advanced glaucoma were statistically older than patients with early glaucoma and than healthy controls. Neverthless BDNF and NGF serum levels were not related to age, neither in the sample population nor in subgroups, allowing us to exclude the age effect as a potential bias of results.

In our study, women had higher serum levels of NGF and BDNF compared to men, both in glaucoma patients and in healthy controls and this finding was already reported in previous studies [41,43].

In the present study there was no difference in IOP levels between the three subgroups of glaucoma patients so we didn’t evaluate the influence of IOP on BDNF and NGF serum levels. It would be interesting to explore in future studies whether topical pharmacological hypotensive treatments have an impact on BDNF and NGF levels. An upregulation of BDNF by brimonidine has already been described in experimental models on rat retinal ganglion cells [44]. In the present study we cannot analyze the influence of brimonidine on NTs serum levels because only two patients in the early stage and four patients in the advanced stage of glaucoma were treated with this drug (Table 1). Of interest, BDNFmRNA upregulation was observed in rats topically treated with Betaxolol but only one patient in our study was treated with this drug [45]. Furthermore another challenge would be to explore the relationship between systemic levels of neurotrophic factors and the risk and rate of glaucoma progression. Moreover, the present study was designed to be focused only on POAG patients but future studies could consider to include other forms of glaucoma, including secondary glaucomas that can serve as controls for disease specificity and potentially give insight into the disease pathophysiology.

In conclusion, according to the results of our study serum levels of NGF and BDNF vary across different stages of glaucoma, suggesting that their levels might be the expression of progressive neurodegenerative damage that occurs over the course of the disease. Nevertheless since NGF and BDNF may be produced also by a variety of non-neural cell types in response to different stimuli, further investigations are required to assess their role as potential biomarkers of glaucoma.

## References

[1] Quigley HA, Broman AT. The number of people with glaucoma worldwide in 2010 and 2020. Br J Ophthalmol 2006;90:262–7. 10.1136/bjo.2005.081224 16488940 PMC1856963

[2] Tham YC. Global Prevalence of Glaucoma and Projections of Glaucoma Burden through 2040. Ophthalmology 2014;121:2081–90. 10.1016/j.ophtha.2014.05.013 24974815

[3] Quigley HA. Neuronal death in glaucoma. Prog Retin Eye Res 1999;18: 39–57. 9920498 10.1016/s1350-9462(98)00014-7

[4] Weinreb R. Primary open-angle glaucoma. Lancet 2004;363:1711–20. 10.1016/S0140-6736(04)16257-0 15158634

[5] Micera A, Quaranta L, Esposito G, Floriani I, Pocobelli A, Saccà SC et al Differential Protein Expression Profiles in Glaucomatous Trabecular Meshwork: An Evaluation Study on a Small Primary Open Angle Glaucoma Population. Adv Ther. 2016;33:252–67. 10.1007/s12325-016-0285-x 26820987 PMC4769730

[6] Pease ME, McKinnon SJ, Quigley HA, Kerrigan-Baumrind LA, Zack DJ. Obstructed Axonal transport of BDNF and its receptor TrkB in experimental glaucoma. Invest Ophthalmol Vis Sci. 2000;41:764–74. 10711692

[7] Quigley HA, McKinnon SJ, Zack DJ, Pease ME, Kerrigan-Baumrind LA, Kerrigan DF et al Retrograde axonal transport of BDNF in retinal ganglion cells is blocked by acute IOP elevation in rats. Invest Ophthalmol Vis Sci. 2000;41:3460–6. 11006239

[8] Almasieh M. The molecular basis of retinal ganglion cell death in glaucoma. Prog Retin Eye Res 2012;31:152–81. 10.1016/j.preteyeres.2011.11.002 22155051

[9] Lewin GR, Barde YA. Physiology of the neurotrophins. Annu Rev Neurosci. 1996;19:289–317. 10.1146/annurev.ne.19.030196.001445 8833445

[10] Gupta V, You Y, Li J, Gupta V, Golzan M, Klistorner A et al BDNF impairment is associated with age-related changes in the inner retina and exacerbates experimental glaucoma. Biochim Biophys Acta. 2014;1842:1567–78. 10.1016/j.bbadis.2014.05.026 24942931

[11] Roberti G, Mantelli F, Macchi I, Massaro-Giordano M, Centofanti M. Nerve growth factor modulation of retinal ganglion cell physiology. J Cell Physiol. 2014;229:1130–3. 10.1002/jcp.24573 24501088

[12] Micera A, Lambiase A, Stampachiacchiere B, Bonini S, Bonini S, Levi-Schaffer F. Nerve growth factor and tissue repair remodeling: trkA(NGFR) and p75(NTR), two receptors one fate. Cytokine Growth Factor Rev. 2007;18:245–56. 10.1016/j.cytogfr.2007.04.004 17531524

[13] Lambiase A, Aloe L, Centofanti M, Parisi V, Báo SN, Mantelli F et al Experimental and clinical evidence of neuroprotection by nerve growth factor eye drops: implications for glaucoma. Proc Natl Acad Sci U S A. 2009 11;106:13469–74. 10.1073/pnas.0906678106 19805021 PMC2726400

[14] Domenici L, Origlia N, Falsini B, Cerri E, Barloscio D, Fabiani C et al Rescue of retinal function by BDNF in a mouse model of glaucoma. PLoS One. 2014;23:9. 10.1371/journal.pone.0115579 PMC4275209 25536045

[15] Poduslo JF, Curran GL. Permeability at the blood-brain and blood-nerve barriers of the neurotrophic factors: NGF, CNTF, NT-3, BDNF. Brain Res Mol Brain Res. 1996;36:280–6. 8965648 10.1016/0169-328x(95)00250-v

[16] Laske C, Stransky E, Leyhe T, Eschweiler GW, Maetzler W, Wittorf A et al BDNF serum and CSF concentrations in Alzheimer’s disease, normal pressure hydrocephalus and healthy controls. J Psychiatr Res. 2007;41:387–94. 10.1016/j.jpsychires.2006.01.014 16554070

[17] Scalzo P, Kümmer A, Bretas TL, Cardoso F, Teixeira AL. Serum levels of brain-derived neurotrophic factor correlate with motor impairment in Parkinson's disease. J Neurol 2010;257:540–5. 10.1007/s00415-009-5357-2 19847468

[18] Ghaffariyeh A, Honarpisheh N, Shakiba Y, Puyan S, Chamacham T, Zahedi F et al Brain-derived neurotrophic factor in patients with normal-tension glaucoma. Optometry 2009;80:635–8. 10.1016/j.optm.2008.09.014 19861219

[19] Ghaffariyeh A, Honarpisheh N, Heidari MH, Puyan S, Abasov F. Brain-derived neurotrophic factor as a biomarker in primary open-angle glaucoma. Optom Vis Sci 2011;88:80–5. 10.1097/OPX.0b013e3181fc329f 21076359

[20] Weskamp G, Otten U. An enzyme-linked immunoassay for nerve growth factor (NGF): a tool for studying regulatory mechanisms involved in NGF production in brain and in peripheral tissues. J Neurochem. 1987;48:1779–86. 3572400 10.1111/j.1471-4159.1987.tb05736.x

[21] Micera A, Lambiase A, Puxeddu I, Aloe L, Stampachiacchiere B, Levi-Schaffer F et al Nerve growth factor effect on human primary fibroblastic-keratocytes: possible mechanism during corneal healing. Exp Eye Res 2006;83:747–57. 10.1016/j.exer.2006.03.010 16716299

[22] Coassin M, Lambiase A, Sposato V, Micera A, Bonini S, Aloe L. Retinal p75 and bax overexpression is associated with retinal ganglion cells apoptosis in a rat model of glaucoma. Graefes Arch. Clin. Exp. Ophthalmol 2008;246:1743–49. 10.1007/s00417-008-0913-5 18751719

[23] Laske C, Stransky E, Leyhe T, Eschweiler GW, Wittorf A, Richartz E et al Stage-dependent BDNF serum concentrations in Alzheimer's disease. J Neural Transm (Vienna) 2006 9;113:1217–24. 16362629 10.1007/s00702-005-0397-y

[24] Faria MC, Gonçalves GS, Rocha NP, Moraes EN, Bicalho MA, Gualberto Cintra MT et al Increased plasma levels of BDNF and inflammatory markers in Alzheimer's disease. J Psychiatr Res 2014;53:166–72. 10.1016/j.jpsychires.2014.01.019 24576746

[25] Angelucci F, Spalletta G, di Iulio F, Ciaramella A, Salani F, Colantoni L et al Alzheimer's disease (AD) and Mild Cognitive Impairment (MCI) patients are characterized by increased BDNF serum levels. Curr Alzheimer Res 2010 2;7:15–20. 20205668 10.2174/156720510790274473

[26] Durany N, Michel T, Kurt J, Cruz-Sánchez FF, Cervás-Navarro J, Riederer P. Brain-derived neurotrophic factor and neurotrophin-3 levels in Alzheimer's disease brains. Int J Dev Neurosci 2000;18:807–13. 11156744

[27] Hellweg R, Gericke CA, Jendroska K, Hartung HD, Cervós-Navarro J. NGF content in the cerebral cortex of non-demented patients with amyloid-plaques and in symptomatic Alzheimer's disease. Int J Dev Neurosci 1998;16:787–94. 10198825 10.1016/s0736-5748(98)00088-4

[28] Manni L, Rocco ML, Bianchi P, Soligo M, Guaragna M, Barbaro SP et al Nerve growth factor: basic studies and possible therapeutic applications. Growth Factors 2013;31:115–122. 10.3109/08977194.2013.804073 23777359

[29] Fahnestock M, Michalski B, Xu B, Coughlin MD. The precursor Pro-Nerve Growth factor Is the Predominant Formo f nerve Growth factor in Brain and Is Increased in Alzheimer’s Disease. Molecular and Cellular Neuroscience 2001; 18: 210–220. 10.1006/mcne.2001.1016 11520181

[30] Schumer RA, Podos SM The nerve of glaucoma! Arch Ophthalmol 1994;112:37–44. 8285890 10.1001/archopht.1994.01090130047015

[31] Aloe L, Bracci-Laudiero L, Alleva E, Lambiase A, Micera A, Tirassa P. Emotional stress induced by parachute jumping enhances blood nerve growth factor levels and the distribution of nerve growth factor receptors in lymphocytes. Proc Natl Acad Sci U S A. 1994;91:10440–4 7937971 10.1073/pnas.91.22.10440 PMC45036

[32] Alleva E, Aloe L, Bigi S. An updated role for nerve growth factor in neurobehavioural regulation of adult vertebrates. Rev Neurosci 1993;4:41–62. 7952382 10.1515/revneuro.1993.4.1.41

[33] Aloe L, Alleva E, Böhm A, Levi-Montalcini R. Aggressive behavior induces release of nerve growth factor from mouse salivary gland into the bloodstream. Proc Natl Acad Sci U S A. 1986;83:6184–7. 3090553 10.1073/pnas.83.16.6184 PMC386464

[34] Qiu M, W ang SY, Singh K, Lin SC. Association between visual field defects and quality of life in the United States. Ophthalmology 201;12:733–40. 10.1016/j.ophtha.2013.09.043 PMC3943627 24342021

[35] Nibuya M, Morinobu S, Duman RS. Regulation of BDNF and trkB mRNA in rat brain by chronic electroconvulsive seizure and antidepressant drug treatments. J Neurosci 1995;15:7539–47. 7472505 10.1523/JNEUROSCI.15-11-07539.1995 PMC6578063

[36] Dwivedi Y, Rizavi HS, Conley RR Roberts RC, Tamminga CA, Pandey GN. Altered gene expression of brain-derived neurotrophic factor and receptor tyrosine kinase B in postmortem brain of suicide subjects. Arch Gen Psychiatry 2003;60:804–15. 10.1001/archpsyc.60.8.804 12912764

[37] Piccinni A, Veltri A, Costanzo D, Vanelli F, Franceschini C, Moroni I et al Decreased plasma levels of brain-derived neurotrophic factor (BDNF) during mixed episodes of bipolar disorder. J Affect Disord. 2015 1 15;171:167–70. 10.1016/j.jad.2014.08.058 25305432

[38] Fanaei H, Khayat S, Kasaeian A, Javadimehr M. Effect of curcumin on serum brain-derived neurotrophic factor levels in women with premenstrual syndrome: A randomized, double-blind, placebo-controlled trial. Neuropeptides 2016 4;56:25–31 10.1016/j.npep.2015.11.003 26608718

[39] Martinowich K, Lu B. Interaction between BDNF and serotonin: role in mood disorders. Neuropsychopharmacology 2008;33:73–83. 10.1038/sj.npp.1301571 17882234

[40] Chen YW, Lin PY, Tu KY, Cheng YS, Wu CK, Tseng PT. Significantly lower nerve growth factor levels in patients with major depressive disorder than in healthy subjects: a meta-analysis and systematic review. Neuropsychiatr Dis Treat. 2015;11:925–33. 10.2147/NDT.S81432 25897228 PMC4389916

[41] Lommatzsch M, Zingler D, Schuhbaeck K, Schloetcke K, Zingler C, Schuff-Werner P et al The impact of age, weight and gender on BDNF levels in human platelets and plasma. Neurobiol Aging 2005;26:115–23. 10.1016/j.neurobiolaging.2004.03.002 15585351

[42] Ziegenhorn AA, Schulte-Herbrüggen O, Danker-Hopfe H, Malbranc M, Hartung HD, Anders D et al Serum neurotrophins—a study on the time course and influencing factors in a large old age sample. Neurobiol Aging 2007;28:1436–45. 10.1016/j.neurobiolaging.2006.06.011 16879899

[43] Trajkovska V, Marcussen AB, Vinberg M, Hartvig P, Aznar S, Knudsen GM. Measurements of brain-derived neurotrophic factor: methodological aspects and demographical data. Brain Res Bull 2007;73:143–9. 10.1016/j.brainresbull.2007.03.009 17499648

[44] Gao H, Qiao X, Cantor LB, WuDunn D. Up-regulation of brain-derived neurotrophic factor expression by brimonidine in rat retinal ganglion cells. Arch Ophthalmol 2002;120:797–803. 12049586 10.1001/archopht.120.6.797

[45] Wood JP, DeSantis L, Chao HM, Osborne NN. Topically applied betaxolol attenuates ischaemia-induced effects to the rat retina and stimulates BDNF mRNA. Exp Eye Res. 2001;72:79–86. 10.1006/exer.2000.0929 11133185

